# Family Socioeconomic Status and Internalizing Problem Behavior Among Chinese Adolescents: The Chain Mediation Effect of Academic Performance and Peer Conflict

**DOI:** 10.3389/fpsyg.2022.902545

**Published:** 2022-06-23

**Authors:** Yangyang Wang, Tian Xie, Jian Xu

**Affiliations:** ^1^China Institute for Urban Governance, Shanghai Jiao Tong University, Shanghai, China; ^2^School of International and Public Affairs, Shanghai Jiao Tong University, Shanghai, China; ^3^School of Media and Communication, Shanghai Jiao Tong University, Shanghai, China

**Keywords:** family SES, internalizing problem behavior, academic performance, peer conflict, urban governance for mental health

## Abstract

This study aims to provide a new perspective on the relationship between family socioeconomic status (SES) and internalizing problem behavior (IPB) among adolescents. Many studies have focused on the relationship between family SES and IPB among adolescents; however, research on the underlying mechanism is still insufficient, and peer conflict has been ignored as a crucial social relationship factor for adolescents. This study identifies two new mediating variables and a chain mediating mechanism model between them. Using national longitudinal data from 2,467 adolescents aged 10–15 published in the China Family Panel Studies of wave 2018, this study found the following: (1) higher family SES can significantly reduce peer conflict and IPB among adolescents; (2) adolescents with better academic performance were less likely to be involved in peer conflict; (3) peer conflict mediated 30.41% of the relationship between family SES and adolescent’s IPB; and (4) there was a chain mediating mechanism, and the mediating effect of peer conflict was much stronger than the mediating effect of both academic performance and the chain mediation pathways. This is the first study to develop a chain mediation model to examine the roles of academic achievement and peer conflict in the relationship between family SES and IPB. These findings are significant in that they highlight the importance of providing adolescents with proper emotional de-escalation and peer conflict resolution strategies, contributing to the management of adolescent mental health in urban governance and rural development.

## Introduction

Internalizing problem behavior (IPB) that contains implicit, inner-directed symptoms (e.g., generalized depression and anxiety) is relatively common during adolescence (Hughes and Gullone, 2008; Graber and Sontag, 2009). Adolescents adolescence is a sensitive period characterized by various social, biological, and psychological changes (Christie and Viner, 2005; Hanson and Chen, 2007). Adolescents may experience strained parent–child relationships and peer conflict, and their mental health can be impacted by these developments. An analysis of 29 studies (including 80,879 adolescents globally) suggested that one in four adolescents have depression symptoms, while one in five adolescents experience anxiety symptoms (Racine et al., 2021). Adolescents in China are also increasingly suffering from mental health issues (Xiong et al., 2017). According to the report on National Mental Health Development in China (2019–2020), almost one in four (24.6%) Chinese adolescents report feeling depressed, and 7.4% of them have severe depressive symptoms (Cynthia, 2022). In general, internalization problems such as depression and anxiety are becoming increasingly prominent in China.

Internalizing problem behavior is associated with diverse negative outcomes, including lack of exercise (Laukkanen et al., 2002), alcohol abuse (Essau et al., 2014), cigarette smoking (Johnson, 2000), sleep problems (Pieters et al., 2015), and suicide behaviors (Lee et al., 2006; Piqueras et al., 2019). Moreover, IPB is likely to extend into adulthood (Pine et al., 1998). Given the adverse effects of IPB on adolescents, many studies have been conducted to explore the predictors of IPB, and family socioeconomic status (SES) has been recognized as an important factor (Mills et al., 2012; Bøe et al., 2014; McNeilly et al., 2021). The relationship between family SES and psychological problems has long been established (Chen, 2004). Studies have indicated that lower family SES predicts higher rates of IPB like depression and anxiety in children and adolescents (Keiley et al., 2000; Gilman et al., 2003; El-Sheikh et al., 2010; Fontaine et al., 2019; McNeilly et al., 2021). However, the underlying mediating mechanism remains unclear and understudied. Furthermore, relevant research in the Chinese context is lacking.

Family SES is defined as a family’s access to multiple forms of economic, human, and social resources, such as household income, parental occupation, and parental education level (Peverill et al., 2021). According to the social causality hypothesis, factors associated with family SES play a role in developmental issues. Multiple components of family SES have been associated with IPB: lower parental education levels (Goodman et al., 2003; Demir et al., 2013), persistent poverty (Slopen et al., 2010), and low parental occupation (van Oort et al., 2011) have all been linked to a higher likelihood of internalized problems. Moreover, each component of family SES can have a different effect on the symptom of internalizing. One study discovered that the mother’s educational attainment is more likely to affect the depression level of female adolescents, while the effect of family income level has a greater impact on the depression level of male adolescents (Zhou et al., 2018). Generally, the relationship between family SES disadvantage and IPB has been widely confirmed in the literature. However, some studies found that there was no significant relationship between family SES and IPB (Barbarin and Richter, 2001; Stevens et al., 2007), and an empirical analysis of Chinese children confirmed this assertion (Jiang et al., 2018). Therefore, further exploration is needed into the effect of family SES on IPB in adolescents and its mechanism of action in the Chinese context.

Furthermore, family SES has been shown to account for most of the differences in academic performance, such as test scores, grade point average, and school dropout rates (Coleman, 2006; Altschul, 2012). A meta-analysis revealed a medium to strong relationship between family SES and academic success (Sirin, 2005). Compared to adolescents of higher SES families, those who came from economically disadvantaged families reported lower levels of intrinsic academic motivation (Manganelli et al., 2021), and lower academic performance (Bae and Wickrama, 2015; Zhang et al., 2020). In addition, poor academic performance has been linked to an increased likelihood of IPB (Bruffaerts et al., 2018). Sörberg Wallin et al. studied 26,766 Swedish individuals and found that academic underachievement is highly associated with depression in young adulthood (Sörberg Wallin et al., 2019). A study of Canadian teens has corroborated that poor academic performance predicts increased IPB for both boys and girls (van Lier et al., 2012). Research has established that family SES has an effect on academic performance, and academic performance predicts IPB. As a result, we inferred that the impact of family SES on IPB is mediated by academic performance.

Peer conflict refers to mutual opposition (e.g., disagreements, verbal disputes, emotional quarrels, and physical fights) between individuals with similar levels of psychological development (Opotow, 1991; Hartup et al., 1993). A systematic review indicated that peer-level factors have not been well explored in the relationship between family SES and negative psychosocial outcomes in adolescents (Devenish et al., 2017). Previous research has shown that family SES is related to adolescent interpersonal conflict; children with lower family SES are less respected and accepted by their peers (Jiang et al., 2018), have fewer friendships (Alivernini et al., 2019), and are more likely to engage in direct verbal and physical violence (Marini et al., 2006; Stalmach et al., 2014; Heshmat et al., 2016). It has been shown that adolescents who frequently argue with peers are more likely to have psychosocial problems (Patterson et al., 1989). Additionally, the conflict aspect of friendship quality has been shown to be positively correlated with internalizing behaviors like depression and anxiety (Bosacki et al., 2007; You and Bellmore, 2012). This evidence supports the hypothesis that peer conflict mediates the association between family SES and internalizing problems.

In light of the existing literature, we hypothesize that academic performance and peer conflict serve in a mediating role between family SES and IPB. However, the existence of a chain effect between the two mediating variables is still to be confirmed. It has been shown that academic achievement and peer relationships are complementary and positively correlated (Wang et al., 2019). On the one hand, peer conflict may hinder adolescents’ academic performance (Goguen et al., 2010). On the other hand, academic performance plays an important role in peer relationships (Palacios et al., 2019). Moreover, educational failure may increase the risk of bullying. Children with poor academic performance can be viewed as a hinderance to their classroom’s goal of educational achievement, leading to frequent victimization by peers (Juvonen and Graham, 2001; Schwartz et al., 2001). In the context of Confucian culture, academic achievement has been regarded as students’ primary task (Chen et al., 2008). Outstanding academic performance not only reflects personal value and family honor, but it can also be regarded as an important aspect of peer relations (Ng et al., 2014). High academic performance enables students to gain recognition and respect from their peers. Hence, we assume that academic performance affects peer conflict in the Chinese context. Generally, academic performance and peer relationships function as chain mediators in the relationship between family SES and IPB.

Numerous studies have shown that family SES is associated with IPB. Based on the family stress model, children from low SES families are at risk of decreased academic success and social competence and increased IPB (Bradley and Corwyn, 2002; Conger and Donnellan, 2007; Conger et al., 2010). According to the family investment model, higher SES families may provide more resources and social capital to their children, which are beneficial to children’s educational success, interpersonal connections, and mental health (Bradley et al., 2001; Conger and Donnellan, 2007; Goosby, 2007). According to Bronfenbrenner’s ecological systems theory, family, neighborhood, peer, and school networks are the main ecosystems for children (Bronfenbrenner, 1986). However, no study has incorporated family SES, academic performance, peer conflict, and IPB into an integrated framework.

**Figure 1:**
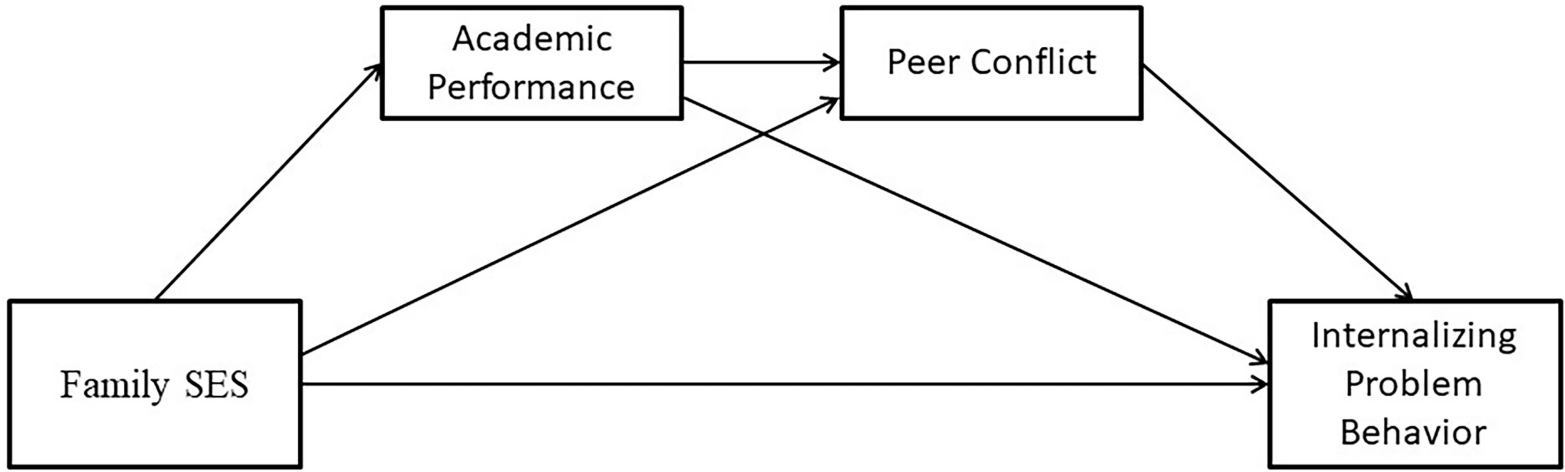
The relationship between family socioeconomic status (SES), internalizing problem behavior (IPB), academic performance, and peer conflict.

To address these knowledge gaps, we examined the pathway of how family SES affects IPB. Four hypotheses have been developed in the hypothesized model (see Figure 1): (1) family SES is negatively related to IPB (Hypotheses 1); (2) academic performance mediates the relationship between family SES and IPB (Hypotheses 2); (3) peer conflict mediates the relationship between family SES and IPB (Hypotheses 3); and (4) both academic performance and peer conflict exhibit a chain mediating effect in the relationship between family SES and IPB (Hypotheses 4).

## Materials and Methods

### Data and Sampling

The data were obtained from the 2018 wave of the China Family Panel Studies (CFPS), which was funded by the 985 Program of Peking University and conducted by the Institute of Social Science Survey of Peking University. This national multidisciplinary social tracking survey covered 25 provincial administrative regions with a target sample size of 16,000 households. The survey population included all household members, and the CFPS 2018 database included 14,241 households. A total of 2,467 participants aged 10–15 were included in this study after removing invalid samples. The sample included 1,322 boys (53.59%) and 1,145 girls (46.41%).

### Measurement

#### Internalizing Problem Behavior

Internalizing problem behavior in adolescents was originally measured using the Achenbach deviant behavior scale (Peterson and Zill, 1986). The CFPS 2018 used a more streamlined version derived from the United States Early Childhood Longitudinal Study, which has eight questions measuring IPB. Each question was measured using a five-degree Likert scale, with a total score of 5–40. In addition, the IPB has a Cronbach’s *α* of 0.68, which fulfills the criteria for further analysis (Peterson, 1994; Cho and Kim, 2015).

#### Family Socioeconomic Status

Three dimensions measured family SES: net family income, parental years of education, and parental occupational prestige; this method has been widely adopted (Haller and Portes, 1973; Buchmann, 2002; Korous et al., 2018). The score for parental occupational prestige was obtained from Treiman’s international prestige scale (Treiman, 2001). The family SES score was formed by principal component analysis (PCA), with a higher score indicating a higher family SES.

#### Academic Performance

Academic performance was measured by a single question: “What was your rank in your grade on the most recent major exam?” Five possible answers were included: “top 10%” (coded 5), “11–25%” (coded 4), “26–50%” (coded 3), “51–75%” (coded 2), and “Post 24%” (coded 1). Higher values indicated greater academic performance. This one-item measurement has been adopted in several studies (Guo et al., 2019a,b, 2021).

#### Peer Conflict

Peer conflict was measured by two questions: “I often quarrel with my peers,” and “I get into trouble for fighting with my peers.” These two questions were measured by using the five-degree Likert scale, with higher scores indicating more frequent occurrences. The average score of the two questions was used to measure peer conflict. One previous study supports this measurement practice (Chung and Fuligni, 2011).

#### Control Variables

Based on previous studies (Cauce et al., 2000; Guo et al., 2018; Jiang et al., 2021; Kang et al., 2021), three categories of control variables related to adolescent internalization were chosen for this study. The first category includes socio-demographic variables, including gender (male, female), age, school stage (primary school, junior high school, high school/junior high school/technical school/vocational high school, and college), whether the participants live at home (yes, no), and whether they have pocket money (yes, no). The second category includes health behavior variables, including whether they smoke (yes, no), whether they drink alcohol (yes, no), and BMI. The third category includes family characteristics variables, including residence (urban, rural), number of quarrels with parents per month (NQWP), household size, and access to the internet (yes, no).

### Statistical Analysis

Using SPSS19 and Stata17, descriptive statistics and categorical statistics based on IPB scores were conducted, as well as a χ^2^ test or one-way ANOVA. The SD and mean were then calculated, along with a correlation analysis among key variables. The bias-corrected percentile bootstrap method was chosen as an appropriate method for testing the chain mediation model (Edwards and Lambert, 2007). With the help of SPSS19 and the PROCESS 4.0 macro, Model 6 was selected and the number of bootstrap samples was set to 5,000 (Hayes, 2015). A significant mediating effect was indicated if the 95% CI did not include zero (Xiao et al., 2022). Finally, robustness tests were performed using SPSS19, including the ordinal logistic model (OLM) and hierarchical linear model (HLM).

## Results

### Descriptive Statistics of Variables

The descriptive statistics of the participants are shown in Table 1. The sample size of boys (53.59%) was greater than that of girls (46.41%), and their average age was 12.39 years old. Rural residents represented 58.69% of the population, compared to 41.31% in urban regions, with the majority in primary school (59.22%) and junior high (39.08%). After the *χ^2^* test or one-way ANOVA, it was found that the frequencies or means of schooling stage (*p* < 0.01), NQWP (*p* < 0.001), family SES (*p* < 0.000), academic performance (*p* < 0.001), and peer conflict (*p* < 0.001) were significantly different in different IPB score groups. Conversely, there was no significant difference in the IPB scores groups for the characteristic variables, such as gender, age, and residence.

**Table 1:** Characteristics of study participants and internalizing problem behavior score distribution (*n* = 2,467).

Variable	Total	IPB(8–15)	IPB(16–19)	IPB(20–23)	IPB(24–40)	*χ*^2^ test/One-way ANOVA	*p*
	*N*(%)	*N*(%)	*N*(%)	*N*(%)	*N*(%)		
Gender, *N* (%)						3.52	0.318
Female	1,145(46.41)	287(45.48)	329(44.58)	271(46.8)	258(49.71)		
Male	1,322(53.59)	344(54.52)	409(55.42)	308(53.2)	261(50.29)		
Age, Mean (*SD*)	12.39(1.66)	12.2(1.62)	12.46(1.67)	12.46(1.65)	12.46(1.7)	0.93	0.335
Residence, *N* (%)						2.77	0.428
Rural	1,448(58.69)	359(56.89)	427(57.86)	343(59.24)	319(61.46)		
Urban	1,019(41.31)	272(43.11)	311(42.14)	236(40.76)	200(38.54)		
Living at home, *N* (%)						1.72	0.632
No	36(1.46)	7(1.11)	12(1.63)	7(1.21)	10(1.93)		
Yes	2,431(98.54)	624(98.89)	726(98.37)	572(98.79)	509(98.07)		
Schooling stage, *N* (%)						22.85	0.007
Primary School	1,461(59.22)	409(64.82)	424(57.45)	327(56.48)	301(58)		
Junior High	964(39.08)	214(33.91)	303(41.06)	245(42.31)	202(38.92)		
HJTV	41(1.66)	8(1.27)	11(1.49)	6(1.04)	16(3.08)		
College	1(0.04)	0(0)	0(0)	1(0.17)	0(0)		
Have pocket money, *N* (%)						4.61	0.202
Yes	1893(76.73)	469(74.33)	563(76.29)	448(77.37)	413(79.58)		
No	574(23.27)	162(25.67)	175(23.71)	131(22.63)	106(20.42)		
Smoking, *N* (%)						0.86	0.836
No	2,454(99.47)	629(99.68)	734(99.46)	575(99.31)	516(99.42)		
Yes	13(0.53)	2(0.32)	4(0.54)	4(0.69)	3(0.58)		
Drinking, *N* (%)						2.65	0.449
No	2,447(99.19)	626(99.21)	733(99.32)	576(99.48)	516(99.42)		
Yes	20(0.81)	5(0.79)	5(0.68)	3(0.52)	3(0.58)		
NQWP, Mean (*SD*)	1.16(3)	1(3.03)	1.01(2.26)	1.16(2.65)	1.59(4.06)	13.44	0.000
Family size, Mean (*SD*)	5.09(1.84)	5.01(1.9)	5.06(1.84)	5.2(1.79)	5.14(1.83)	0.34	0.559
Internet access						7.31	0.063
No	1,471(59.63)	242(38.35)	292(39.57)	226(39.03)	236(45.47)		
Yes	996(40.37)	389(61.65)	446(60.43)	353(60.97)	283(54.53)		
BMI, Mean (*SD*)	18.58(3.75)	18.43(3.8)	18.58(3.55)	18.82(3.92)	18.52(3.78)	0.2	0.651
Family SES, Mean (*SD*)	0(0.94)	0.11(0.94)	0.05(0.98)	−0.02(0.95)	−0.18(0.87)	25.17	0.000
Academic performance, *N* (%)						35.24	0.000
Top 10%	331(13.42)	97(15.37)	109(14.77)	77(13.3)	48(9.25)		
11–25%	384(15.57)	101(16.01)	121(16.4)	90(15.54)	72(13.87)		
26–50%	1,338(54.24)	360(57.05)	388(52.57)	311(53.71)	279(53.76)		
51–75%	290(11.76)	51(8.08)	84(11.38)	70(12.09)	85(16.38)		
Post 24%	124(5.03)	22(3.49)	36(4.88)	31(5.35)	35(6.74)		
Peer conflict, Mean (*SD*)	1.78(0.8)	1.42(0.62)	1.71(0.68)	1.88(0.73)	2.22(1)	417.25	0.000
IPB, Mean (*SD*)	19.14(5.24)	12.83(2)	17.52(1.11)	21.32(1.1)	26.68(2.87)		
Total	2,467	631(25.58)	738(29.91)	579(23.47)	519(21.04)		

HJTV, High School/Junior High School/Technical School/Vocational High School; NQWP, Number of quarrels with parents per month; BMI, Body Mass Index; and IPB, the score of internalizing problem behavior.

### Correlation Analysis of Variables

Bivariate correlations between the core variables were conducted by using Pearson correlation analysis. The results are presented in Table 2, including the mean values, SDs, and Pearson correlation values of the core variables. The results indicated that IPB was positively associated with peer conflict (*r* = 0.38, *p* < 0.001), but negatively associated with family SES (*r* = −0.11, *p* < 0.001) and academic performance (*r* = −0.1, *p* < 0.001). Moreover, family SES was positively associated with academic performance (*r* = 0.1, *p* < 0.001) but negatively associated with peer conflict (*r* = −0.1, *p* < 0.001). Finally, academic performance was negatively associated with peer conflict (*r* = −0.11, *p* < 0.001).

**Table 2:** Correlation analysis between key variables (*n* = 2,467).

	Mean	*SD*	Internalizing problem behavior	Family SES	Academic performance	Peer conflict
Internalizing problem behavior	19.14	5.24	–			
Family socioeconomic status	0.00	0.95	−0.11^***^	–		
Academic performance	3.21	0.98	−0.1^***^	0.1^***^	–	
Peer conflict	1.78	0.8	0.38^***^	−0.1^***^	−0.11^***^	–

*** *p* < 0.001.

### Chain Mediating Effects Analysis

**Figure 2:**
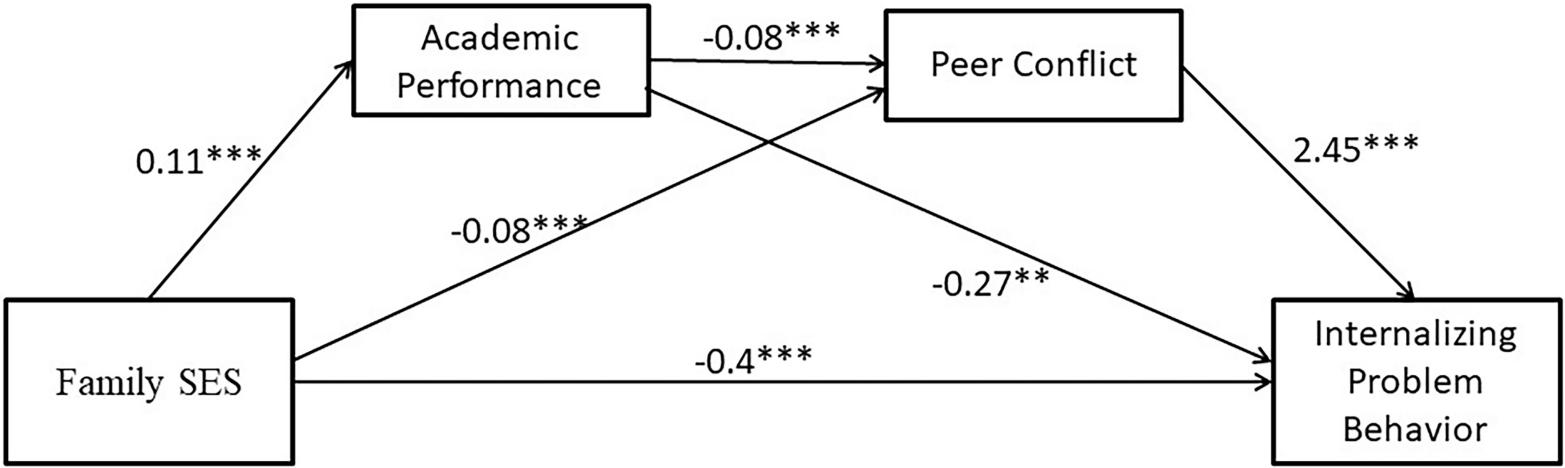
The chain mediating effect of academic performance and peer conflict. **p < 0.01 and ***p < 0.001.

After introducing the three types of control variables mentioned in the “Measurement” section above, a test for mediating effects was conducted using PROCESS 4.0. It should be noted that prior to the regression analysis, we performed a multicollinearity test on the variables and the VIF values were all less than 5, in line with the current specification. The results are presented in Table 3 and Figure 2. In Equation 1, Family SES positively affected academic performance (*b* = 0.03, *p* < 0.001). In Equation 2, peer conflict was negatively influenced by family SES (*b* = −0.08, *p* < 0.001) and academic performance (*b* = −0.08, *p* < 0.001). In Equation 3, family SES (*b* = −0.4, *p* < 0.001) and academic performance (*b* = −0.27, *p* < 0.01) negatively influenced IPB, while peer conflict positively influenced IPB (*b* = 2.45, *p* < 0.001). In Equation 4, family SES (*b* = −0.65, *p* < 0.001) negatively influenced IPB, supporting Hypothesis 1.

**Table 3:** Regression results of the chain mediating effects model (*n* = 2,467).

Outcome variable	Predictive variable	*R* ^2^	*F*	*b*	SEs	*t*	LLCI	ULCI
Equation 1								
Academic performance	Family SES	0.03	4.93^***^	0.11^***^	0.02	4.91	0.068	0.158
Equation 2								
Peer conflict	Family SES	0.06	9.92^***^	−0.08^***^	0.02	−4.33	−0.117	−0.044
	Academic performance			−0.08^***^	0.02	−5.17	−0.116	−0.052
Equation 3								
Internalizing problem behavior	Family SES	0.17	31.09^***^	−0.4^***^	0.11	−3.48	−0.622	−0.174
	Academic performance			−0.27^**^	0.1	−2.75	−0.47	−0.078
	Peer conflict			2.45^***^	0.12	19.78	2.204	2.69
Equation 4								
Internalizing problem behavior	Family SES	0.28	5.08^***^	−0.65^***^	0.12	−5.298	−0.889	−0.409

All estimated coefficients are unstandardized. Control variables of the regression model include gender, residence, living at home, schooling stage, have pocket money, smoking, drinking, NQWP, family size, internet access, and BMI. LLCI, Lower limit of the 95% CI, and ULCI, Upper limit of the 95% CI; and Number of bootstrap samples for percentile bootstrap confidence intervals is 5,000.

** *p* < 0.01 and

*** *p* < 0.001.

The results of the mediating effects analysis showed that academic performance and peer conflict mediated the relationship between family SES and IPB (see Figure 2). Specifically, there were four paths through which family SES influenced IPB: (a) Family SES → Internalizing problem behavior; (b) Family SES → Academic performance → Internalizing problem behavior; (c) Family SES → Peer conflict → Internalizing problem behavior; and (d) Family SES → Academic performance → Peer conflict → Internalizing problem behavior. Thus, path (d) proves the existence of the chain mediating effect mechanism, supporting Hypotheses 2–4.

### Total Effect, Direct Effect, and Indirect Effect of the Chain Mediating Effect

After confirming the chain mediating effect, the total, direct, and indirect effects of the chain mediating effect were calculated, as shown in Table 4. The results showed that the total indirect effect (−0.25) accounted for 38.75% of the total effect (−0.65) and 63.28% of the direct effect (−0.4) in the relationship between family SES and IPB. This result indicates that 38.75% of the negative effect that family SES exerts on IPB works through three mediating effects. Specifically, the effects are (a) the mediating effect of academic performance, (b) the mediating effect of peer conflict, and (c) the mediating effects of academic performance and peer conflict, respectively. The mediating effects (a), (b), and (c) represented 4.76, 30.41, and 5.38% of the total effect, respectively, and 7.78, 49.66, and 5.84% of the direct effect, respectively. The mediating effect of peer conflict was significantly stronger than academic performance or chain mediation in the relationship between family SES and IPB. In addition, all the above tests of the total effect, direct effect, and indirect effect, and mediating effects of (a), (b), and (c) were statistically significant at 95% CIs that did not overlap with zero.

**Table 4:** Results and comparison of chain mediating effect (*n* = 2,467).

	Effect	Boot *SE*	Boot LLCI	Boot ULCI	Ratio of indirect to total effect	Ratio of indirect to direct effect
Total effect	−0.65	0.12	−0.889	−0.409	–	–
Direct effect	−0.4	0.11	−0.622	−0.174	–	–
Total indirect effect	−0.25	0.05	−0.347	−0.164	38.75%	63.28%
Ind1	−0.03	0.01	−0.059	−0.008	4.76%	7.78%
Ind2	−0.2	0.05	−0.289	−0.113	30.41%	49.66%
Ind3	−0.02	0.01	−0.038	−0.011	3.58%	5.84%

Ind1 is the mediation effect model of Family SES → *Academic performance* → *Internalizing problem behavior, Ind2 is the mediation effect model of Family SES* → *Peer conflict* →*Internalizing problem behavior, and Ind3 is the mediation effect model of Family SES* → *Academic performance* → *Peer conflict* → Internalizing problem behavior. Boot SE, Boot LLCI, and Boot ULCL are estimated SE under bias-corrected percentile bootstrap method, and 95% CI lower and 95% CI upper, and Boot LLCI and Boot ULCL do not overlap with zero.

### Robustness Test

The validity and consistency of the research findings were assessed using robustness tests. Based on the database, we opted to conduct the robustness test by substituting the model, recoding the variables, and conducting subsample regressions. Prior to the test, IPB was coded as a four-category variable (code “8–15” as “1”; code “16–19” as “2”; code “20–23” as “3”; and code “24–40” as “4”). The higher the value, the more severe the internalized problem behavior.

To rule out confounding variables, we re-tested the overall sample (OLM 1), the female sample (OLM 2), and the male sample (OLM 3) by using the ordinal logistic model (OLM) and the recoded dependent variable, as shown in Table 5. The findings indicated that family SES continued to have a negative impact on IPB (*p* < 0.01).

**Table 5:** Results of robustness tests.

Variables	Full Sample	Female Sample	Male Sample	Full Sample	Full Sample
	OLM 1	OLM 2	OLM 3	HLM Model 1	HLM Model 2
Family SES	−0.209^***^	−0.264^***^	−0.158^**^	−0.639^***^	−0.617^***^
	(0.040)	(0.058)	(0.056)	(0.124)	(0.122)
Control variable	–	–	–	–	–
	–	–	–	–	–
Wald chi2	63.08	20.21	26.64	69.17	69.45
Pseudo *R*^2^	0.009	0.016	0.005	0.000	0.000
Log pseudolikelihood	−3365.63	−1557.22	−1800.79	−7554.75	−7552.18
Observations	2,467	1,145	1,322	2,467	2,467

Control variables of the regression model include gender, residence, living at home, schooling stage, have pocket money, smoking, drinking, NQWP, family size, internet access, and BMI. Robust SEs in parentheses.

** *p* < 0.01 and

*** *p* < 0.001.

A further test was required, as the adolescents in this study were clustered within families. This multilevel structure had the potential to affect the SE and model fitting in cases when the intraclass correlation is not trivial. We used the hierarchical linear model (HLM) to perform a robustness test to mitigate the potential impact of this data structure. HLM Model 1 and Model 2 represent the random intercept and random coefficient models, respectively. Table 5 demonstrates that family SES has a continuing negative impact on IPB (*p* < 0.001). Table 3 shows that the regression results are valid and stable, indicating that our findings are well supported.

## Discussion

Based on the national cross-sectional data from the 2018 wave of CFPS, this study validates the underlying mechanism between family SES and IPB in Chinese adolescents. More specifically, this study suggests that family SES significantly and negatively predicts IPB in adolescents; this finding is consistent with previous studies (Najman et al., 2010; Devenish et al., 2017). Furthermore, in accordance with our assumptions, this study indicates that family SES indirectly influences IPB *via* academic performance, peer conflict, and the chain mediating effect of academic performance and peer conflict. The total mediating effect is 38.75%, which shows a critical influence of mediators on the relationship between family SES and IPB.

This study finds that academic performance mediates the association between family SES and IPB (with a mediating effect of 7.78%). This finding indicates that adolescents from lower SES families are more likely to experience IPB when performing poorly in academics. Consistent with previous research, this study finds family SES positively associated with academic performance (Bradley and Corwyn, 2002; Li et al., 2020), with poor academic performance as a risk factor for IPB (Moilanen et al., 2010). Compared with lower-SES families, higher-SES families may provide more resources and social capital which can help students succeed in school (Coleman, 1988; Conger and Donnellan, 2007). As academic achievement is regarded as the sole criterion for a Chinese student’s success (Yu et al., 2006). Poor academic performance may predict a high level of IPB like depression and anxiety (Li and Zhang, 2008; Zeng et al., 2021). It is worth noting that Chinese culture emphasizes the importance of academic achievement (Chen et al., 2008). This finding suggests that more diverse ways of evaluating student achievement should be employed in the Chinese context.

As expected, this study strongly supports the hypothesis that peer conflict mediates the relationship between family SES and IPB (with a mediating effect of 30.41%). Therefore, peer conflict is a key mediator between family SES and IPB. Our findings support earlier research showing that lower family SES is related to peer conflict (Heshmat et al., 2016; Korous et al., 2018), and peer conflict is a risk factor for IPB (Biggs et al., 2010). In general, adolescents with low family SES may be more likely to engage in peer conflict which can lead to IPB. One study has shown that effective peer conflict resolution can reduce negative emotions (Wang et al., 2014). Therefore, solving conflicts rationally instead of confrontationally may buffer the relationship between low family SES and high levels of IPB.

Furthermore, this study illustrates the chain mediating effect of academic performance and peer conflict between family SES and IPB. In other words, adolescents with lower family SES are more likely to develop IPB through lower academic performance and a higher level of peer conflict. A previous study has confirmed the relationship between family SES and academic performance, showing that academic performance has both direct and indirect negative effects on subsequent peer victimization and depression (Liu et al., 2018). Concretely speaking, higher academic performance reduces the level of subsequent depression by reducing peer victimization (Shen, 2020). A possible explanation for this finding is that underachievers may experience more contempt and less friendship, leading to peer conflict. Moreover, adolescents from low SES families face educational inequality, which may result in higher levels of peer conflict and internalization.

Finally, our findings support the ecological systems theory that microsystems, such as family, school, and peers play an important role in adolescent mental health. In addition, previous research has typically defined the boundaries and participants of microsystems (Neal and Neal, 2013). Our study illustrates that the boundaries between these microsystems are fuzzy, different microsystems interact with each other and work together in adolescent development. Family microsystem, which is associated with other microsystems, plays a crucial role in the development of adolescent mental health.

## Limitations

There are several limitations to this study. First, a cross-sectional survey was employed, and the causative interpretations cannot be determined. For example, a bidirectional association between academic performance and peer conflict. The clarification of whether academic performance and peer conflicts are a cause or a consequence requires future longitudinal studies. Second, this study lacked a standard measurement of peer conflict and academic performance; more scientific measurements should be used in future studies. Third, the distinct Chinese cultural background that emphasizes education may affect the validity and stability of these findings in different cultural contexts. Fourth, adolescents were nested within schools and families; this multilevel structure has implications for standard errors and model fit when the intraclass correlation is not evident. Lastly, we could not rule out the influence of potential confounders on the study findings due to data limitations. Parental psychopathology is one of the potential confounding factors of family economic status and adolescent mental health (Reiss, 2013). Migration background is also an important potential confounder (Alivernini et al., 2020; Manganelli et al., 2021). It is necessary to statistically control for these potential confounders in further studies.

Despite these limitations, this study has several strengths. First, this is the first study to examine the mediating role of peer conflict in the relationship between family SES and IPB. Second, many previous studies have shown that peer conflict affects academic performance, and this study provides evidence that the reverse association exists. This finding suggests that good academic performance helps to reduce the impact of low family SES on poor peer relationships. Still, for those from low SES families, problems such as educational inequality and interpersonal conflicts deserve the attention of governments, schools, and families. Third, these findings provide some inspiration: efforts should be made at multiple micro-levels (e.g., family, peer, and individual) to reduce the problem of internalization and build a better mental health environment for adolescents.

## Conclusion

This study examined the mediating role of academic performance and peer conflict between family SES and IPB in adolescents. The findings show that family SES is negatively associated with IPB directly and indirectly through academic performance and peer conflict. In addition, this study examined the following mediating pathway: Family SES → Academic performance → Peer conflict → Internalizing problem behavior. Based on these findings, it is necessary to develop policies to prevent IPB in adolescents. To achieve this goal, interventions that target low academic achievement and peer conflict rather than low socioeconomic status would be more effective. Therefore, the Chinese government should increase educational investments for adolescents with low family SES to promote educational equity in urban governance and rural development. Additionally, appropriate psychological counseling services would be beneficial for adolescent peer conflict resolution.

## Data Availability Statement

The raw data supporting the conclusions of this article will be made available by the authors, without undue reservation.

## Ethics Statement

This study was conducted based on de-identified, publicly available CFPS data and did not interact with any individuals or use identifiable private information. Therefore, the ethics statement was waived.

## Author Contributions

Study concept and design and drafting of the manuscript were contributed by YW and TX. Acquisition, analysis, and interpretation of data were contributed by YW. Critical revision of the manuscript for important intellectual content was contributed by YW, TX, and JX. Funding acquisition and supervision were contributed by JX. All authors contributed to the article and approved the submitted version.

## Funding

This research was funded by the National Social Science Foundation of China’s key research project, grant number 17AXW002.

## Conflict of Interest

The authors declare that the research was conducted in the absence of any commercial or financial relationships that could be construed as a potential conflict of interest.

## Publisher’s Note

All claims expressed in this article are solely those of the authors and do not necessarily represent those of their affiliated organizations, or those of the publisher, the editors and the reviewers. Any product that may be evaluated in this article, or claim that may be made by its manufacturer, is not guaranteed or endorsed by the publisher.
